# Cell penetrating caspase substrates promote survival of the transplanted cells

**DOI:** 10.1186/s13104-019-4480-0

**Published:** 2019-07-19

**Authors:** Andrey Mikhailov, Yoshiyuki Sankai

**Affiliations:** Center for Cybernics Research, University of Tsukuba, Tsukuba, Japan

**Keywords:** Apoptosis, Cell penetrating peptides, Caspase, Cleavage, Internalization, Cell transplantation

## Abstract

**Objective:**

Cell survival in critical post-transplantation period is challenged by inflammation, lack of vascularization, and insufficient cell attachment anchoring. Temporally blocking cell death may increase cell survival, but it is important to possess no risks of sustained cell death signal blocking and possible malignant transformations. Regarding apoptotic cell death, multi-micromolar overloading the cell with competitive caspase substrates delays the effects of actual downstream enzyme activation processing. Later, when introduced substrate is consumed, and the caspase activation stimuli may still be present, the apoptotic cell death can proceed normally.

**Results:**

Here we studied several synthetic peptides comprising from effector caspase activational cleavage sequences fused with various internalization motifs. Designed peptides showed rapid and efficient internalization into cultured neuroblast cells comparing to non-fused cleavage sequences as measured by cytofluorimetry and confirmed by mass spectrometry. Pretreatment with selected peptides protected the cells from several apoptogenic stimuli in vitro, as well as improved survival of syngeneic immortalized Schwann cells during transplantation in vivo.

## Introduction

Regeneration and reconnection of the injured nerves are still challenging tasks despite the stunning progress in both material development and micro-surgical procedures. Transplantation of the stem cells and/or supporting cells expected to provide a natural way of functional recovery, however, low cell viability after the transplantation decreased the success rate of such attempts in the past [1]. First several days after the transplantation is a critical time for survival, since the cells have not yet established their attachment points to the extracellular matrix, injury site is infiltrated with immune cells producing inflammatory cytokines, and no vascularization in the area had been started yet leading to low local oxygen concentration. Many cells at such conditions die by apoptosis, anoikis, or necrosis. Apoptotic cell death is mediated by activation of the specific class of proteases called caspases. They cleave a wide range of protein substrates inside the cell, preparing it to die without causing the inflammatory response in cell’s surrounding. Caspases are present in cells as inactive zymogenes and become activated by different macromolecular interactions, the most prominent of which is the activation by cleavage from others (upstream) caspases. If we supply the cell with an excess of the initiator caspase substrates, the cleavage of natural substrates and the further activation of the caspase cascade will be delayed. It would allow transplanted cells necessary time for adaptation to the environment at the site of transplantation. Peptides are short-lived cell-degradable molecules, which (1) can be tailored to mimic exact structure of the caspase substrates/cleavage sites and (2) their internalization inside the cells can be facilitated by fusion with known functional sequences. For our study we chose two cell lines resembling neuronal (neuroblast, N1E) and supportive (Schwann cells, R3) cells.

## Main text

### Methods

The following peptides (see abbreviations section for sequence information) and their C-terminal FITC derivatives were synthesized using FMOC-chemistry: CPEP, LL31, LL18, DL18, 3196.

Direct FACS analysis of FITC-labelled peptides. About 10^6^ N1E (ATCC^®^ CRL2263™) cells were exposed to 100 nM of peptides with terminally attached FITC in normal culture media (DMEM/RPMI1640 mixed in ratio 1/1 with 10% of fetal calf serum) for 10 min at 37 °C. After incubation cell were washed twice with phosphate-buffered saline (PBS), detached by Trypsin-5.3 mmol/l EDTA solution and fixed in 4% paraformaldehyde for 30 min at room temperature. Fixed cells were subjected to FACS analysis (LSR, BectonDickenson).

About 10^6^ cells (N1E cells) were exposed to 500 nM of DL18 peptide in normal culture media for 30 min at 37 °C. After exposure the cells were washed twice with PBS, lyzed with 1% Triton X-100 in distilled water and the proteins were sedimented with equal volume of 40% trichloroacetic acid. After centrifugation (9000*g*, 20 min) 20 μl of supernatant was applied on MALDI plate, mixed with solution of 2,5-dihydroxybenzoic acid and subjected for MALDI-TOF analysis.

N1E and R3 ([33-10ras3] (ATCC^®^ CRL-2764™)) cells were grown to about 80% of confluency in 6-well plates covered with d-poly-lysine. Peptides (500 nM or 10 µM) were added to the cells 1 h before treatments. The tested apoptogenic treatments were: Staurosporine (100 nM, 18 h), serum withdraw (20 days for N1E cells and 5 days for R3 cells), heat shock (43 °C, 1 h plus overnight recovery), Camptothecin (10 μM, 18 h), H_2_O_2_ (100 μM, 18 h). After the treatment the cells were carefully washed with PBS and stained simultaneously with Calcein A and Ethidium homodimer-1 (LIVE/DEAD^®^ Viability/Cytotoxicity Kit, LifeTechnologies) for 30 min at room temperature. Cells were photographed in an inverted microscope immediately after staining. Percentage of survived cells was calculated from 3 randomly selected fields of view; average cell death rate ± SEM was calculated using GraphPad Prism (version 6.0 h for MacOS, GraphPad Software, La Jolla, USA). Antibodies specific to cleaved Caspase-3 (Asp175) (Rabbit monoclonal 5A1E, CST) were used to visualize Caspase-3 activation in N1E cells by Western blotting.

We have labelled R3 cells with GFP expressing vector (CellLight™ Nucleus-GFP, BacMam 2.0, Invitrogen™) overnight, collected 10^8^ fluorescent cells by subsequent trypsinization and centrifugation, and divided them into two equal aliquots. One of the aliquots was mixed with the peptide DL18 (10 μM) for 5 min. Then both groups of cells were separately incorporated into ex tempore prepared chitosan/PSTP scaffolds [2] and injected subcutaneously into flank fat of 6 Wistar rats. Two weeks later, the rats were sacrificed by carbon dioxide inhalation in their home cages. The scaffold material was recovered, weighted and analysed by fluorescent microscopy (low magnification, 10×) for density of the fluorescent areas.

### Results

#### Peptides cell penetration

**Fig. 1:**
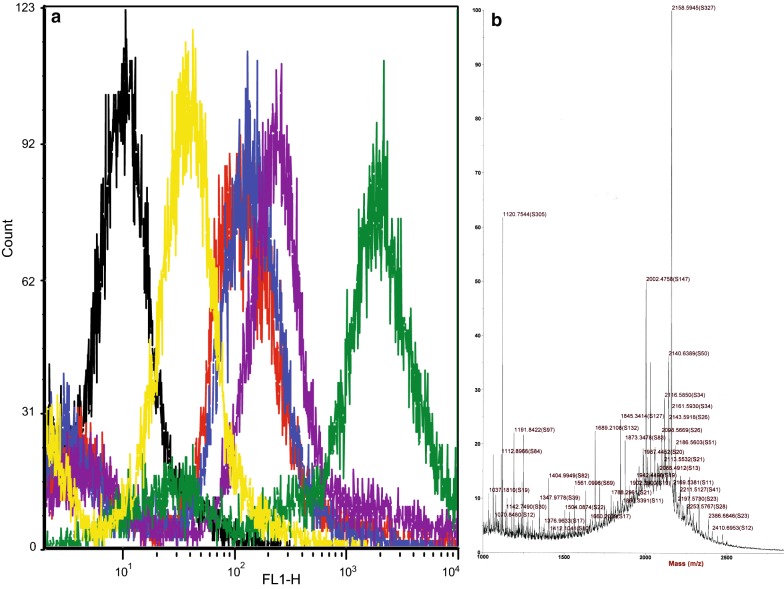
Cellular intake of the tested peptides. **a** FITC-labelled peptides (red—CPEP, blue—LL31, purple—LL18, green—DL18, yellow—3196, black—no peptides) internalize into N1E cells with different efficacy; flow cytometry histogram of fluorescent intensity distribution. **b** Low molecular mass (1–3 kDa) profile of the cell lysates. Target molecular mass 2158 Da (m/z) is detectable among other unknown compounds

From all tested FITC-conjugated peptides the peptide 3196 (lacking cell penetrating motif) showed the lowest cell incorporation (median fluorescence 32.2). Peptides LL18 and DL18 stained cells with the strongest fluorescent signal (Median fluorescence values 212.9 and 1669.77 respectively) (Fig. 1a). These results pointed to the for most active molar incorporation (or faster dynamics of incorporation) and, in either case, defined our choice of the peptides for further study.

Mass spectrometric (MALDI TOF) measurements of trichloroacetic acid-treated cell lysates from peptide loaded cells reveal the strongest peak of m/z 2158 which corresponds to the protonated mass of the non-metabolized DL18 peptide (Fig. 1b). Although our MALDI TOF analysis did not allow exact quantitative comparison between the peaks, the prevalence of the non-metabolized DL18 peptide’s m/z pointed that the dynamics of cellular catabolism is not considerable on 30 min exposure.

#### Effects of the peptides on development of apoptosis

Apoptosis induced by Staurosporine. Staurosporine is an inhibitor of enzyme PKC and a blocker of the survival pathways. N1E cells are highly sensitive to this drug: overnight exposure to 100 nM Staurosporine lead to high degree of cell death (89.67 ± 5.04%) in these cells.

**Fig. 2:**
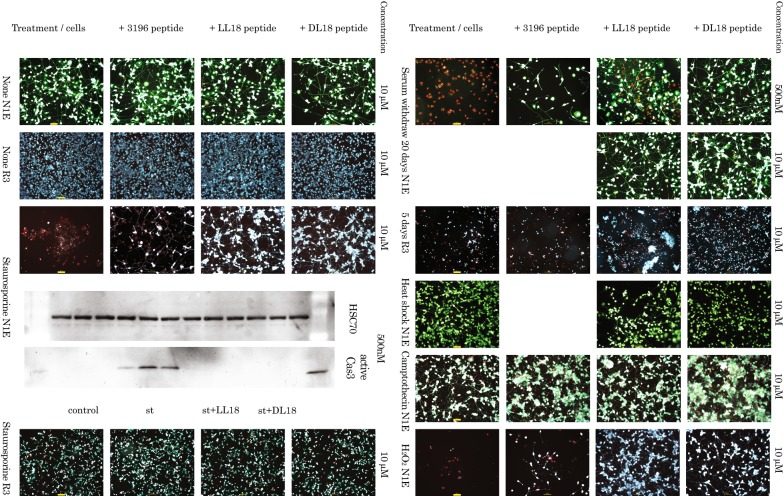
Effect of the peptides DL18 and LL18 (in comparison to commercially available peptide inhibitor 3396) on N1E and R3 cells under different apoptogenic stimuli. Rows 1–3 (left) and 6–12 (right) are fluorescent microscopy (×10 objective; superimposed scale bars are 100 µm) photographs after double staining with Calcein A and Ethidium homodimer. Rows 4–5—Western blotting: Upper line—loading control checked with antibodies against HSC-70, Lower line—caspase cleavage detected with primary antibodies against active caspase 3. Treatments: untreated control, Staurosporine (100 nM), serum withdraw (20 and 5 days), heat shock (43 °C,1 h), Camptothecin (10 μM) and H_2_O_2_ (100 μM)

Both LL18 and DL18 peptides at tested concentrations provided significant protection from development of the cell death (2.67 ± 0.88% and 3.33 ± 1.45% respectively) in N1E cells (Fig. 2, line 3). Alternatively, active Caspase-3, a marker of apoptosis was assessed by Western Blotting analysis of the lysates from neuronal N1E cells exposed to 100 nM Staurosporine in the presence of the two peptides at the above described conditions (Fig. 2, line 5); both LL18 and DL18 peptides efficiently blocked activational cleavage of caspase 3. The sensitivity of R3 cells to Staurosporine was moderate (15.33 ± 3.76% of dead cells), so the protective effects of the developed peptides (13.17 ± 4.08% for LL18 and 12.8 ± 0.75% for DL18) were only marginal (Fig. 2, line 5).

Apoptosis induced by acute serum withdraw. N1E cells are relatively viable at gradual serum withdraw and undergo both differentiation and cell death at that conditions [3]. However, acute serum withdraw pushes majority of the cells in population toward apoptosis (92.6 ± 6.43% of dead cells at day 20). Withdraw of the growth factors is one of the main challenges during first days after cell transplantation. Peptides we investigated proved themselves efficient against it in (26.3 ± 5.36% for LL18 and 20.6 ± 2.91% for DL18, both at 500 nM) dose-dependent manner (Fig. 2, lines 6–7).

R3 cells are sensitive to acute serum withdraw and usually undergo cell death during first several days after treatment starts (79.5 ± 4.54% of dead cells at day 5). Peptides LL18 and DL18 were effective on inducing death retardation after serum withdraw in these cells (24.0 ± 3.46% for LL18 and 16.7 ± 1.93% for DL18, both at 10 µM) (Fig. 2, lines 8).

Heat shock is able to induce apoptosis including activation of the Caspase-2 [4]. The pretreatment of the cells with caspase substrates may benefit the survival after heat shock. However, differentiated neuronal cells are relatively stable to mild heat shock, so the protective effect of the peptides in our tests on this model was marginal (Fig. 2, line 9).

Camptothecin is inhibiting DNA-replicating enzyme topoisomerase I. It is able to block cell division and used as both experimental anti-cancer drug and as apoptosis-inducing agent [5]. Although in our experiments it induces apoptosis in limited number of cells in a population, the peptides did not offer significant protection at tested conditions (Fig. 2, line 10).

Treatment of cells with hydrogen peroxide (H_2_O_2_) mimics free oxygen radical species exposure after the transplantation. Although the exact molecular mechanisms of apoptosis induction by hydrogen peroxide (H_2_O_2_) are not know and, likely, multiple [6], involvement of the caspases was clearly demonstrated [7]. In our experimental setup the peptides DL18 (12.10 ± 4.19% of dead cells) and especially LL18 (only 8.4 ± 0.97% of dead cells) showed high level of protection against the hydrogen peroxide induced apoptosis (84.8 ± 4.28% of dead cells untreated with peptides) (Fig. 2, line 11).

#### Effects of the peptides on transplantation efficiency

**Fig. 3:**
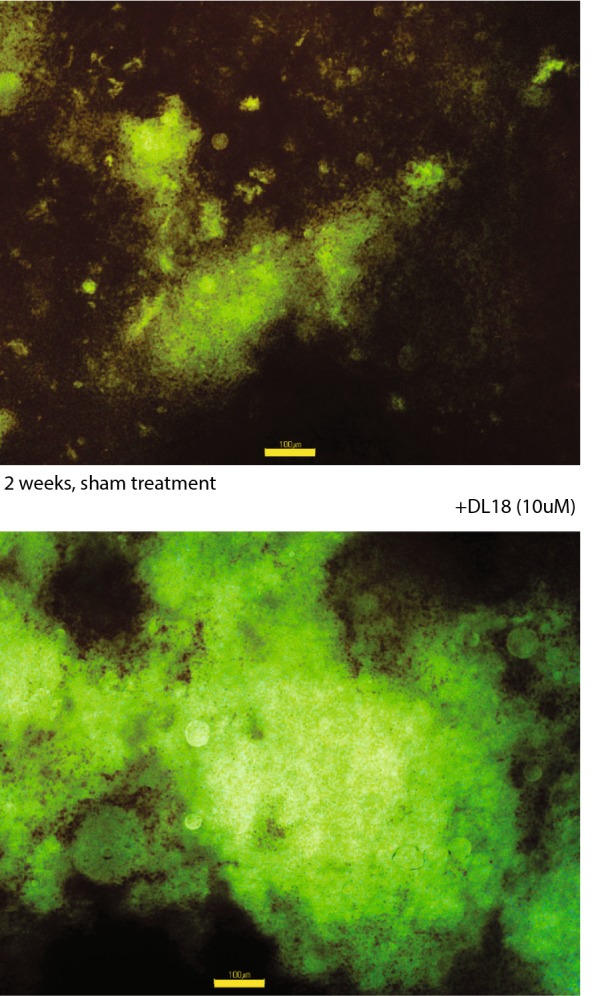
Cell masses 2 weeks after implantation (representative set of photographs at ×10; superimposed scale bars are 100 µm). Cells pretreated with DL18 peptides (10 μM) survives better inside the implanted matrix giving the brighter fluorescence

Following the fate of transplanted cells is a challenging task, so we utilized syngeneic cells which can be transplanted without immune suppression and labelled them with GFP. Survival of the transplanted cells depends on multitude of factors including inflammation at the site of transplantation, limited oxygen supply, problems finding suitable attachment points. In our study average (± SEM) extracted cell masses 2 weeks after implantation for naïve GFP-labelled cell and the same cells pretreated with DL18 peptide (10 μM) were 1.4 ± 0.9 and 7.0 ± 3.1 g. Fluorescence of the transected cell mass was also brighter in case of DL18 pretreated cells (Fig. 3). Together, our results suggest that peptide-pretreated cells survive better inside the implanted matrix giving both the brighter fluorescence and higher cell mass.

### Discussion

Tested peptides demonstrated efficient cell internalization and offered significant protection against cell death caused by growth/survival signaling deficiency (Staurosporine and serum withdraw treatments) and by oxidative stress on two distinct cellular models in vitro. Pretreatment of the cells with the designed peptides before the implantation in animals also increased the survival of the cells.

We expect that the cell permeable peptide caspase substrates improve the outcome of the cell-based transplantation applications in biology and medicine when limited term retardation of the programmed cell death is desirable, including transplantation of stem cells and supportive cells. Importance of preserving some caspase activity during cell transplantation of cardiomyocyte-generating stem cells was stressed recently [8]. Approaches using peptide caspase inhibitors for protection of transplanted organs or selected cell populations were suggested earlier [9], although most of the available results came from utilizing the irreversible peptide [10] or non-peptide caspase inhibitors [11, 12]. Another possible line of applications could include protecting cells viability strait after injury delaying cell death in order to achieve temporary preservation of their physiological function until proper recovery intervention could be performed.

## Limitations

No dynamic internalization studies were performed for peptide cellular uptake making impossible to distinguish between faster dynamics of peptide internalization and maximal tolerable saturation.

Preparation of dying cells for quantitative analysis by microscopy may be difficult because dying cells typically detach from their substrate [13]. This fact makes quantitative assessment of apoptosis on the microphotographs inexact and survival biased.

White balance of the images obtained from the fluorescent microscope was set to automatic and was not preserved between series of image acquisition. Although it did not impact distinguishing between dead and live cells, color of the Calcein A-positive cells ranging from lime to teal on presented images.

## Data Availability

The images and flow cytometry primary files acquired by authors and/or analyzed during the current study will be available from the corresponding author on request.

## Abbreviations

LL18: Gly-Arg-Lys-Lys-Arg-Arg-Gln-Arg-Arg-Arg-Gly-Ile-Glu-Thr-Asp-Ser-Gly-Thr representing activational cleavage sequence for Caspase-3 fused with truncated sequence of TAT protein
DL18: dThr-Gly-dSer-dAsp-dThr-dGlu-dIle-Gly-Arg-Lys-Lys-Arg-Arg-Gln-Arg-Arg-Arg-Gly—representing retro-inverse activational cleavage sequence for Caspase-3 fused with truncated sequence of TAT protein
CPEP: Ser-Ser-Val-Glu-Thr-Asp-Gly-Lys-Glu-Thr-Trp-Trp-Glu-Thr-Trp-Trp-Thr-Glu-Trp-Ser-Gln-Pro-Lys-Lys-Lys-Arg-Lys-Val—representing cleavage sequence for Caspase-8 fused with sequence Pep-1
LL31: Gly-Trp-Thr-Leu-Asn-Ser-Ala-Gly-Tyr-Leu-Leu-Gly-Lys-Ile-Asn-Leu-Lys-Ala-Leu-Ala-Ala-Leu-Ala-Lys-Lys-Ile-Leu-Ser-Glu-Val-Asp-Ser—representing cleavage sequence for Caspase-9 fused with Transportan sequence
3196: Ac-Ile-Glu-Thr-Asp-H (aldehyde)—commercially available non cell penetrable inhibitor for procaspase-3 cleaving enzymes (Caspase-8/6 and Granzyme B)
MALDI TOF: matrix-assisted laser desorption/ionization time of flight
PSTP: penta sodium triphosphate
FACS: fluorescence-activated cell sorting
FITC: fluorescein isothiocyanate
PKC: protein kinase C
GFP: green fluorescent protein
